# Polarization Differential Visible Light Communication: Theory and Experimental Evaluation †

**DOI:** 10.3390/s20195661

**Published:** 2020-10-03

**Authors:** Jorik De Bruycker, Willem Raes, Stanislav Zvánovec, Nobby Stevens

**Affiliations:** 1 KU Leuven, ESAT-DRAMCO, 9000 Ghent, Belgium; willem.raes@kuleuven.be (W.R.); nobby.stevens@kuleuven.be (N.S.); 2 Department of Electromagnetic Field, Faculty of Electrical Engineering, Czech Technical University in Prague, Technicka, 16627 Prague, Czech Republic; xzvanove@fel.cvut.cz

**Keywords:** differential, polarization, Visible Light Communication, quadrant photodiode

## Abstract

Visible Light Communication (VLC) has received substantial research attention in the last decade. The vast majority of VLC focuses on the modulation of the transmitted light intensity. In this work, however, the intensity is kept constant while the polarization direction is deployed as a carrier of information. Demodulation is realized by using a differential receiver pair equipped with mutually orthogonal polarizers. An analytical expression to evaluate the Signal-to-Noise Ratio (SNR) as a function of the rotation angle of the receiver is derived. It is demonstrated that the signal quality can deteriorate heavily with receiver orientation when using a single differential receiver pair. A way to overcome this drawback using two receiver pairs is described. The analytical expression is experimentally verified through measurements with two different receiver setups. This work demonstrates the potential of polarization-based modulation in the field of VLC, where receiver rotation robustness has been achieved by means of a dedicated quadrant photodiode receiver.

## 1. Introduction

As the need for communication bandwidth increases persistently [1], Visible Light Communication (VLC) has emerged as an alternative means for wireless communication. VLC capitalizes on the omnipresence of light-emitting diodes (LEDs) and exploits their ability to modify the transmitted intensity at high data rates in order to modulate signals in the visible light spectrum [2]. Several physical layer implementations for VLC have been included in IEEE 802.15.7 [3]. VLC has shown to be a promising development for both indoor and outdoor communications. Indoor applications also include indoor positioning systems [4,5]. In recent work, quadrature photodiodes equipped with dedicated apertures were employed to provide indoor localization based on Angle-of-Arrival (AoA) [6,7]. The communication properties of such receiver have also been investigated in detail [8].

Rather than intensity based modulation in VLC, an additional interesting opportunity lies in modulating the polarization state of visible light. For instance, PIXEL [9] uses a polarizer in conjunction with a Liquid Crystal (LC) to modulate the polarization axis of light at low data rates. Demodulation is realized by means of a polarizer-equipped camera receiver. The data rate is however severely limited by the response time of the LC and the camera refresh rate. POLI [10] also uses a camera equipped with a polarizer but combines the polarizers with a dispersor at the transmitter side to alter the perceived color at the camera. Here also, the data rate is also constrained by the low camera update rate. Additionally, orthogonal polarizers have been employed as a multiplexing technique [11], referred to as polarization-division multiplexing (PDM). Researchers demonstrated an optimized pre-equalization circuit [12] and proposed an asymmetric 3 × 2 multi-input multi-output (MIMO) system for PDM [13]. Recent studies have applied PDM in conjunction with Orthogonal Frequency Division Multiplexing (OFDM), achieving a 45% increase in data rate and spectral efficiency [14]. Related work demonstrated a setup consisting of red/green/blue laser diodes reaching 40 Gbit/s using PDM OFDM signals [15]. Furthermore, other research has used orthogonal polarizers at both the transmitter and the receiver side to implement differential signalling between polarization states on one hand and to increase robustness to interference on the other hand [16]. These systems deploy photodiode receivers, which increase the bandwidth significantly compared to camera-based receivers. While the receiver orientation was fixed in aforementioned work [16], the impact of the receiver rotation with respect to the transmitters was assessed in previous work [17]. In this work, this assessment is reiterated and extended. The presented polarization-based modulation scheme is functional alongside intensity-based approaches, meaning optical bandwidth can be shared among both approaches. An analytical expression is derived to express the impact of receiver rotation. This dependency is also assessed in a representative indoor scenario and compared with the theoretical elaboration. It is demonstrated that the signal quality can deteriorate heavily with receiver orientation when using a single differential receiver pair. A second differential receiver pair can be deployed to overcome this drawback. This method is experimentally evaluated using a custom design based on a quadrant photodiode. The contents of this work is structured as follows: Section 2 describes the general system model, modulation scheme and corresponding signal processing. An experimental setup used to verify this model is presented in Section 3, while the measurement results are given in Section 4. Finally, the main conclusions of this work can be found in Section 5.

## 2. System Model

### 2.1. Polarization

As light is an electromagnetic wave, the electric and magnetic fields oscillate in a certain direction perpendicular to the propagation direction. For most light sources, including LEDs, this oscillation varies randomly over time, this is referred to as unpolarized light. If the electric and magnetic fields however oscillate in a fixed plane, it is said that the light is linearly polarized. In this work, linear polarizers (LPs) are used in order to transmit light with linear polarization. An ideal LP has a defined axis along which polarized light is unaltered. Light polarized orthogonally to this axis is either reflected or absorbed, depending on the type of polarizer. Unpolarized light passing through a LP is thus polarized along the axis of the LP. When linearly polarized light traverses a polarizer, the intensity *I* in the transmission is given by (1), generally known as Malus’ Law [18]. Here $I_{0}$ is the intensity incident on the polarizer and ϕ is the angle between the polarization axis of the incident light and the polarizer axis:

(1) $$I=I_{0}\cos^{2}\left(\phi \right).$$

### 2.2. Modulation and Demodulation

**Figure 1:**
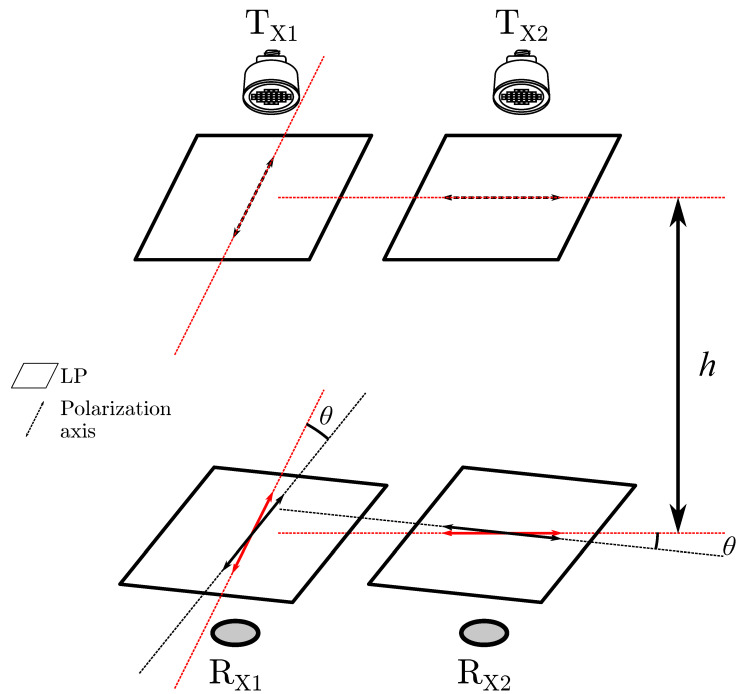
Setup consisting of two LEDs and two photodiodes, each equipped with mutually orthogonal linear polarizers (LP) [17].

In order to transmit data using linearly polarized light, the intensity of the transmitted light is kept constant, but the polarization axis is rotated orthogonally in order to represent ‘1’ and ‘0’ bits. To achieve this in practice, a setup using two identical LED transmitters $T_{X1}$ and $T_{X2}$ is constructed where both LEDs are equipped with mutually orthogonal linear polarizers as indicated in the upper part of Figure 1. The LEDs are power switched in a complementary way, i.e., LED $T_{X1}$ is on while $T_{X2}$ is off during a bit period $T_{b}$, thus emitting polarized light along the axis of the $T_{X1}$ LP to represent a ‘1’-bit. Vice versa, turning LED $T_{X1}$ off whilst turning $T_{X2}$ on emits light polarized along an axis orthogonal to the first case, effectively representing a ‘0’-bit. Following this mode of operation, the information is thus completely embedded in the polarization direction of the transmitted light. This modulation scheme can also be considered to be a polarization-based variant of On-Off Keying (OOK).

Demodulation at the receiver can be effectively realized using a similar setup. A pair of photodiodes, $R_{X1}$ and $R_{X2}$, are likewise equipped with mutually orthogonal polarizers as indicated in the lower part of Figure 1. The differential signal between this pair of photodiodes can then be used to reconstruct the transmitted signal at the transmitter. As the LEDs are transmitting an identical intensity in a complementary fashion, the received intensity is constant over time if no polarizing optics were equipped, thus limiting interference with conventional intensity-based VLC and inherently preventing human-perceivable flicker. The receiver polarizers maintain mutual orthogonal alignment by the design of the receiver, but unlike the transmitter polarizers, the orientation of the receiver polarizers is not fixed and can rotate over an angle θ with respect to the transmitter polarizers axes. At $\theta =0^{\circ }$ the polarizer at $T_{X1}$ is parallel with the $R_{X1}$ polarizer while at $T_{X2}$, the polarizer is parallel with the $R_{X2}$ polarizer. In this setup, the distance between transmitter and receiver is denoted as *h*, as illustrated in Figure 1.

### 2.3. Signal Processing and Signal-to-Noise Ratio (SNR)

**Figure 2:**
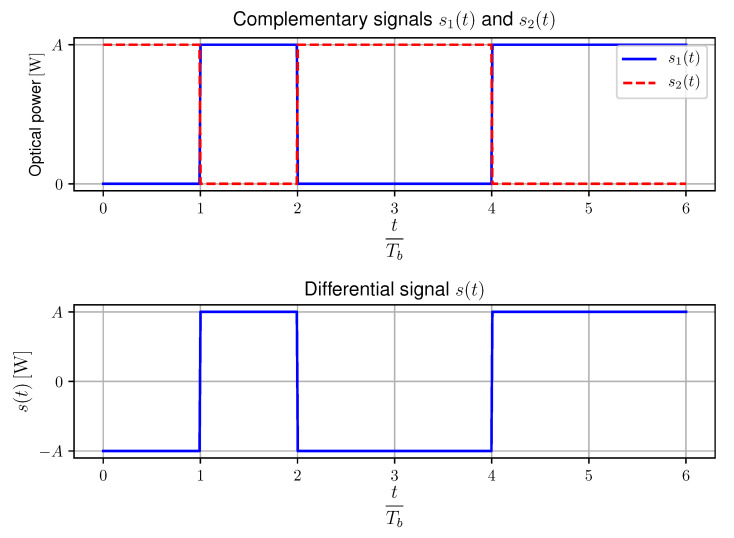
Illustration of ideal complementary transmitted signals $s_{1}\left(t\right)$ and $s_{2}\left(t\right)$ (top) and DC-filtered differential signal s(t) (bottom) as defined by Equation (3) [17].

In order to evaluate the impact of receiver rotation on the SNR, we assume a single receiver pair using a pair of orthogonal polarizers as previously elaborated. The linearly polarized optical signals transmitted by $T_{X1}$ and $T_{X2}$, thus after transmittance through the polarizers, are denoted as $s_{1}\left(t\right)$ and $s_{2}\left(t\right)$ respectively. As mentioned before, $T_{X1}$ and $T_{X2}$ are complementarily toggled resulting in polarization-based modulation. Ideally, the optical power in transmittance *A* is identical for these complementary signals as illustrated in the upper part of Figure 2.

Taking the optical channel model and receiver polarizers into consideration, it can be shown that the received signals at the photodetectors are given by [19]:

(2) $$\begin{array}{cc} \left[\begin{array}{c} r_{1}\left(t\right)\\ r_{2}\left(t\right) \end{array}\right] & =R_{p}\left[\begin{array}{cc} \alpha_{11}\cos^{2}\left(\theta \right) & \alpha_{21}\cos^{2}\left(\theta +90^{\circ }\right)\\ \alpha_{12}\cos^{2}\left(\theta +90^{\circ }\right) & \alpha_{22}\cos^{2}\left(\theta \right) \end{array}\right]\left[\begin{array}{c} s_{1}\left(t\right)\\ s_{2}\left(t\right) \end{array}\right]+\left[\begin{array}{c} w_{1}\left(t\right)\\ w_{2}\left(t\right) \end{array}\right]. \end{array}$$

Here $R_{p}$ is the photodiode responsivity, assumed identical for both photodiodes, $\alpha_{ij}$ is the optical channel gain from $T_{\mathrm{Xi}}$ to $R_{\mathrm{Xj}}$. θ is the rotation angle of the receiver polarizer axes with regard to the transmitter polarizers axes. $w_{1}\left(t\right)$ and $w_{2}\left(t\right)$ are the noise contributions at each photodetector that can be modeled as Additive White Gaussian Noise (AWGN) [20]. These contributions are a sum of thermal and shot noise. While the transmitted signals $s_{1}\left(t\right)$ and $s_{2}\left(t\right)$ typically contain noise contributions as well, these noise contributions can be neglected compared to the thermal and shot noise contributions.

Taking into account the differential signalling operation of $s_{1}\left(t\right)$ and $s_{2}\left(t\right)$, s(t) is defined as:

(3) $$s\left(t\right)=s_{1}\left(t\right)-s_{2}\left(t\right).$$

As shown in the bottom part of Figure 2, s(t) can also be expressed as a signal proportional to the AC-component of $s_{1}\left(t\right)$ and $s_{2}\left(t\right)$:

(4) $$\begin{array}{cc} s(t) & =2\left[s_{1}\left(t\right)-\langle s_{1}\left(t\right)\rangle \right]\\ \; & =-2\left[s_{2}\left(t\right)-\langle s_{2}\left(t\right)\rangle \right], \end{array}$$

where   denotes the mean operator, thus corresponding to the DC-components of $s_{1}\left(t\right)$ and $s_{2}\left(t\right)$. Ideally, the mutual distances between both transmitters and receivers are very small, meaning the difference in path length and the difference in both irradiance and incidence angle are negligible. Under these circumstances, it can be assumed that $\alpha =\alpha_{11}=\alpha_{12}=\alpha_{21}=\alpha_{22}$. Combining Equations (2) and (3) and applying $\cos\left(\theta \pm 90^{\circ }\right)=\mp \sin\left(\theta \right)$ and $\cos\left(2\theta \right)=\cos^{2}\left(\theta \right)-\sin^{2}\left(\theta \right)$, the differential signal r(t) between the photodetectors is thus given by:

(5) $$r\left(t\right)=r_{1}\left(t\right)-r_{2}\left(t\right)=\alpha R_{p}s\left(t\right)\cos\left(2\theta \right)+w\left(t\right),$$

where $w\left(t\right)=w_{1}\left(t\right)-w_{2}\left(t\right)$. As both noise components $w_{1}\left(t\right)$ and $w_{2}\left(t\right)$ consist of differential and common-mode noise, the differential setup effectively suppresses the common-mode noise component by differentiating between the two photodetectors. The differential operation also ensures that interference from intensity-based VLC is eliminated as the contributions of unpolarized, modulated light has an equal impact on both $r_{1}\left(t\right)$ and $r_{2}\left(t\right)$. In practice, r(t) is also DC-filtered to account for any possible asymmetry between the detectors. r(t) is thus to be considered to be a binary antipodal non-return-to-zero coded signal, representing a ‘1’ -bit by a rectangular pulse with a positive amplitude proportional to *A* during a bit period $T_{b}$, and a ‘0’ -bit with an identical negative amplitude, resulting in:

(6) $$r\left(t\right)=\alpha R_{p}A\mathrm{sgn}\left(s\left(t\right)\right)\cos\left(2\theta \right)+w\left(t\right).$$

Several definitions have been used to define the SNR in the field of VLC [21]. In this work, a general approach for antipodal signals is elaborated. The SNR is maximized by applying a matched filter (MF) [22], where the matched filter output *r* is given by:

(7) $$r=\int_{T_{b}}r\left(t\right)s\left(t\right)dt=\mathrm{sgn}\left(s\left(t\right)\right)A^{2}T_{b}\alpha R_{p}\cos\left(2\theta \right)+A\int_{T_{b}}w\left(t\right)dt.$$

Each sample of the matched filter output corresponds to a sample of a Gaussian random variable *R* with mean equal to:

(8) $$\mu_{R}=\mathrm{sgn}\left(s\left(t\right)\right)A^{2}T_{b}\alpha R_{p}\cos\left(2\theta \right),$$

and variance given by:

(9) $$\begin{array}{cc} \mathrm{Var}\left[R\right] & =E\left[{\left(R-\mu_{R}\right)}^{2}\right]\\ \; & =\sigma_{R}^{2}=E\left[{\left(A\int_{T_{b}}w\left(t\right)dt\right)}^{2}\right]. \end{array}$$

The SNR can subsequently be defined as:

(10) $$\mathrm{SNR}=\frac{\mu_{R}^{2}}{\sigma_{R}^{2}}=\frac{{\left(A^{2}T_{b}\alpha R_{p}\cos\left(2\theta \right)\right)}^{2}}{E\left[{\left(A\int_{T_{b}}w\left(t\right)dt\right)}^{2}\right]}.$$

The relation between the SNR and the receiver rotation with respect to transmitter polarization axis θ can thus be written as:

(11) $$\mathrm{SNR}={\mathrm{SNR}}_{0}\;\cos^{2}\left(2\theta \right),$$

where $SNR_{0}$ equals the maximum SNR under the present channel gain with ideal receiver alignment with respect to the transmitter polarization axes and is given by:

(12) $${\mathrm{SNR}}_{0}=\frac{{\left(A^{2}T_{b}\alpha R_{p}\right)}^{2}}{E\left[{\left(A\int_{T_{b}}w\left(t\right)dt\right)}^{2}\right]}.$$

**Figure 3:**
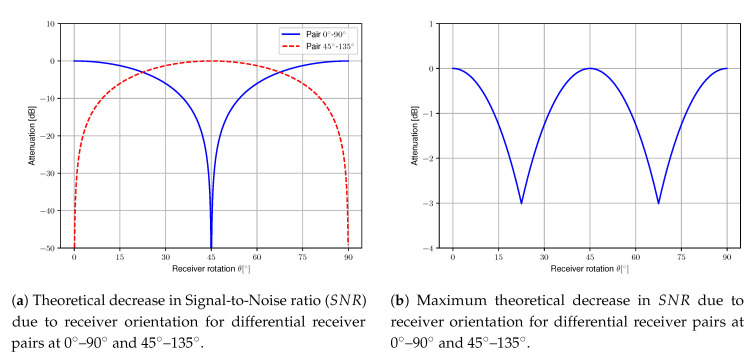
Theoretical decrease in SNR due to receiver orientation for two differential receiver pairs.

As $SNR_{0}$ is by definition independent of the receiver rotation, the factor $\cos^{2}\left(2\theta \right)$ can be considered to be a decrease in SNR dependent on receiver rotation. This function is plotted in Figure 3a. Please note that this dependency varies sharply at $\theta =45{\;}^{\circ }$, hence a minor rotation has a huge impact on the SNR near $\theta =45{\;}^{\circ }$. This can be intuitively explained as the polarizers at $45^{\circ }$ can make no distinction between polarized light originating from $T_{X1}$ and $T_{X2}$ as the angle between the polarization axes is identical. In order to overcome this drawback, an additional differential receiver pair can be placed with polarization axes at $45^{\circ }$ and $135^{\circ }$ for $\theta =0{\;}^{\circ }$. Analogous to the derivation of Equation (11), it can be shown that the SNR for this additional pair is equal to:

(13) $$\mathrm{SNR}={\mathrm{SNR}}_{0}\;\cos^{2}\left(2\left(\theta +45^{\circ }\right)\right).$$

This ensures that at least one pair can receive the transmitted signal without severe attenuation due to receiver orientation. The maximum decrease due to receiver orientation is in this case reduced to −3 dB at $22.5^{\circ }$ and $67.5^{\circ }$ as shown in Figure 3b, or more generally at $\left(2k+1\right)\cdot \frac{45^{\circ }}{2}$ with k∈Z.

As the noise was modeled as AWGN, the determined SNR can be used to determine the Bit-Error Rate (BER) according to [20,22]:

(14) $$BER=Q(\sqrt{SNR}).$$

In this equation, the *Q*-function is given by

(15) $$Q\left(x\right)=\frac{1}{\sqrt{2\pi }}\int_{x}^{\infty }e^{-y^{2}/2}dy.$$

## 3. Measurement Setup

### 3.1. Transmitter Side

**Figure 4:**
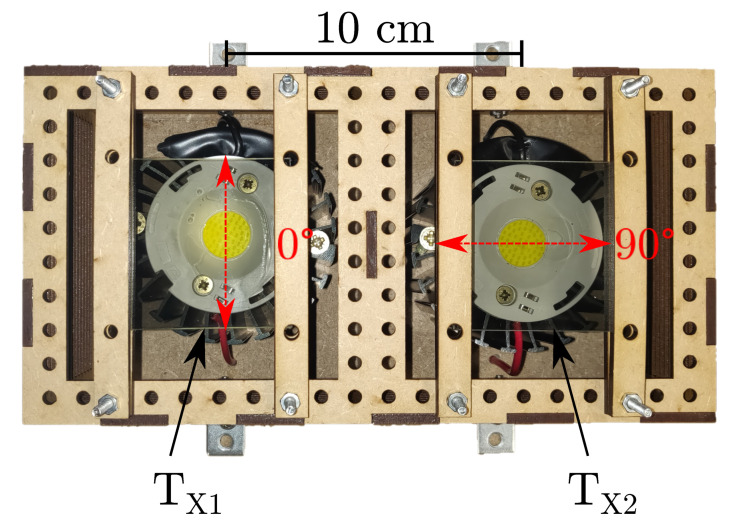
The two LED transmitters equipped with mutually orthogonal polarizers at $0^{\circ }$ and $90^{\circ }$ respectively [17].

The transmitter side consists of two power LEDs (Bridgelux BXRE-50C3001-D-24, Bridgelux, Fremont, CA, US) driven at 300 mA. The LED currents are controlled using two separate drivers (Analog Devices DC2257A, Analog Devices, Norwood, MA, US). Both LEDs are equipped with identical 50 × 50 mm linear plastic polarizers (Edmund Optics 50 × 50 mm Linear Plastic Polarizer XP42-200, Edmund Optics, Barrington, NJ, US) with a specified tolerance on the polarization axis of $\pm 2^{\circ }$. The polarization axes of these polarizers are aligned orthogonally to one another. The transmitter construction is shown in Figure 4, where the polarization axes of the linear polarizers are indicated.

### 3.2. Receiver Side

**Figure 5:**
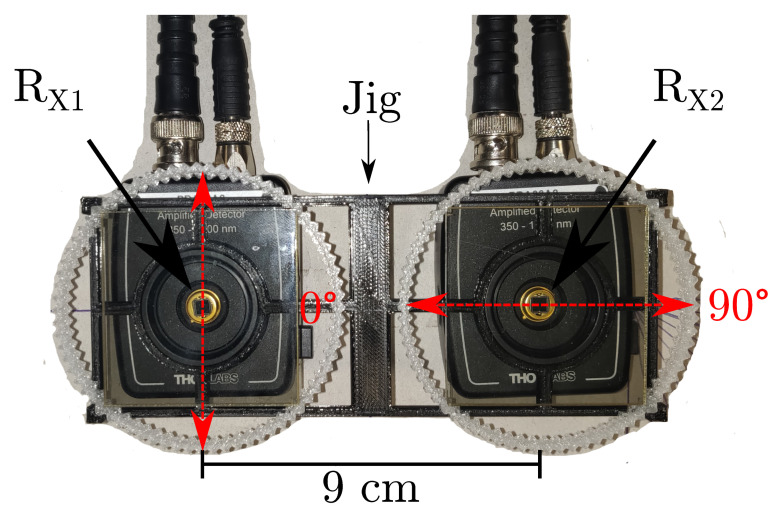
Setup A: two PDA36A2 receivers are equipped with mutually orthogonal polarizers on $5^{\circ }$ increment measurement jig for $\theta =0^{\circ }$ [17].

At the receiver side, two cases are considered. The first case, further mentioned as setup A, uses off-the-shelf available hardware to provide an initial evaluation. Two switchable gain Si detectors (Thorlabs PDA36A2, Thorlabs, Newton, NJ, US) are deployed, also equipped with mutually orthogonal polarizers. These polarizers are rotated in 5 degrees increments using a 3D-printed jig to mimic receiver rotation. Figure 5 shows the assembly of setup A for the case where $\theta =0^{\circ }$. While the mutual distance between transmitters and receivers is ideally negligible, there is still a spacing of 10 cm and 9 cm, respectively between transmitters and receivers in this setup. To minimize the difference in optical path length and irradiance and incidence angle, the receivers are oriented symmetrically relative to the transmitters. The polarizer axes are precisely aligned using a self-leveling crosshair laser (Bosch GLL 3-80, Bosch, Gerlingen, Germany). The setup resembles an indoor environment where h=1.35m.

**Figure 6:**
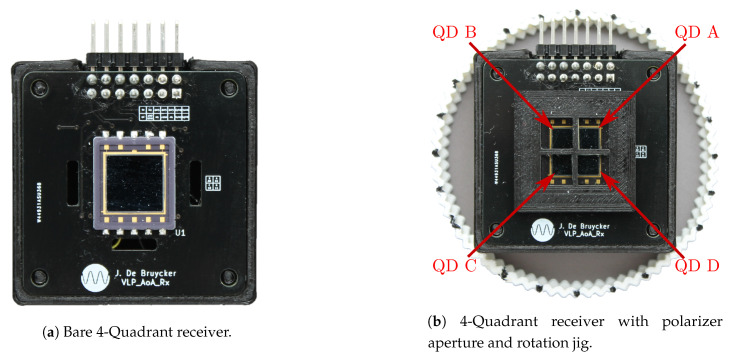

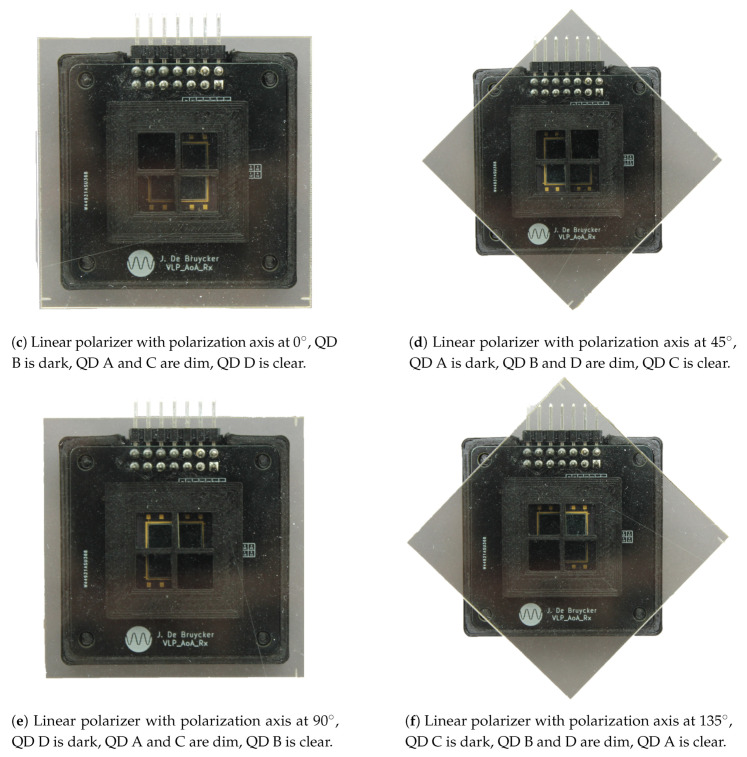
Setup B: four quadrant receiver with aperture containing four linear polarizers subsequently rotated over $45^{\circ }$, note the orientation of the linear polarizing film at each quadrant.

Additionally, a custom receiver was designed for setup B. The receiver is based of a four quadrant (QD) photodiode (Hamamatsu S5981, Hamamatsu, Hamamatsu, Japan) and is shown in Figure 6a. An aperture holding four custom-cut linear polarizing film (Edmund Optics 150 × 150 mm Linear Polarizing Film (XP42-18), Edmund Optics, Barrington, NJ, US) is constructed in front of each quadrant, as shown in Figure 6b. The tolerance of this polarizing film is also specified at $\pm 2^{\circ }$. The height and size of the aperture are designed as such not to obstruct the field of view of the quadrants. The polarization axes of the polarizing film at each quadrant are orientated at angles of $0^{\circ }$, $45^{\circ }$, $90^{\circ }$ and $135^{\circ }$, thus creating two pairs of mutually orthogonal polarizers. Pair 1 consists of quadrants QD B and D with polarizers at $90^{\circ }$ and $0^{\circ }$ respectively while pair 2 is made up of QD A and C with polarizers at $135^{\circ }$ and $45^{\circ }$. The polarization axes of the polarizers at each quadrant are illustrated in Figure 6c–f. The receiver is also rotated in 5 degrees increments with a corresponding jig.

### 3.3. Signal Processing

**Figure 7:**
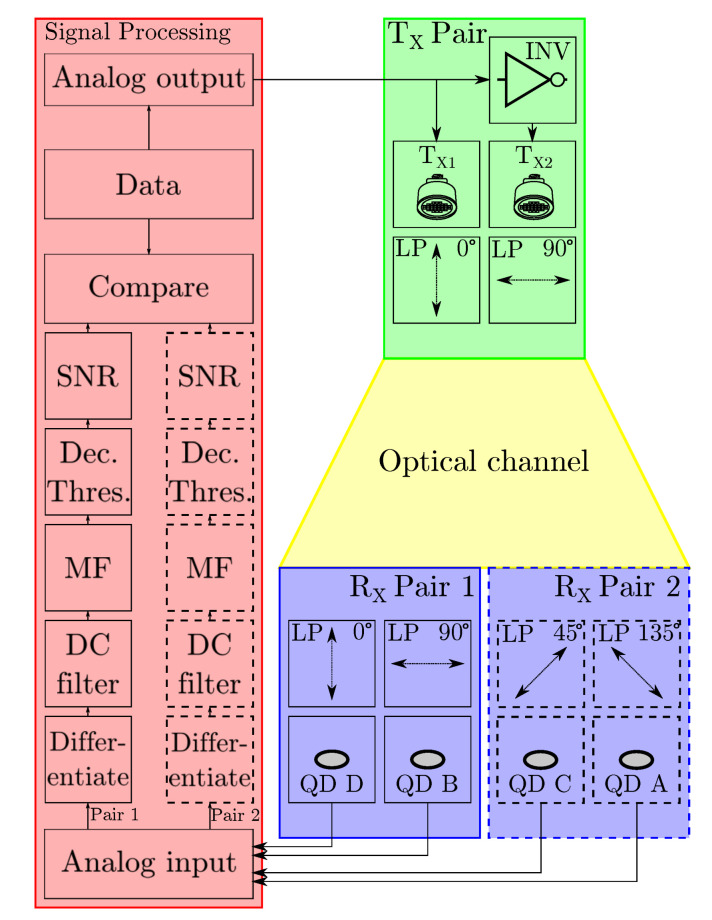
Block diagram illustrating the signal processing chain of both setups. Blocks indicated in striped lines only apply to setup B.

In setup A and B, the LED transmitter switching voltages are controlled by a National Instruments USB-6215 Data Acquisition System (DAQ) and USB-6212, respectively in order to transmit data at 4 kbps. While the polarization differential modulation scheme allows for higher data rates, the data rate in this setup is mainly limited by the used hardware, namely the transient response of the power LEDs and corresponding drivers, and the DAQs used to sample the receivers. The data rate is thus deliberately chosen low as not to disfigure the measurement results and to assess the operating principle accurately. The same DAQ oversamples the photodetector voltages synchronously at 80 kHz. The differential signal is then calculated and DC-balanced to ensure the antipodality of the signal, taking into account any possible asymmetry between the LED transmitters and the receiver photodetectors. This results in a binary antipodal non-return-to-zero coded signal as described in Section 2.3. This signal is then demodulated by matched filtering and based on a decision threshold, the output is mapped back to data bits. As the signal can be inverted depending on the receiver orientation, the data bits can be inverted too. This is accounted for by using a known training sequence at the start of the data transfer to determine whether or not bits should be inverted. The output is then further examined to determine the SNR and compared to the transmitted data to check for bit errors. The key parameters of used photodetectors and DAQ settings for both setups are listed in Table 1. A block diagram illustrating the signal processing chain used for both setups is depicted in Figure 7.

## 4. Measurement Results

The performance of both setups is experimentally evaluated where 8B10B encoding is applied [2]. This encoding has the advantage that the probability of transmitting a ‘1’-bit is equal to the probability of transmitting a ‘0’ -bit, which implies the matched filter output *r* has its optimal decision threshold at the averaged expectation values of the matched filter outputs [22]. The transmitted data consists of an arbitrary text file of 150 kbit. This amount of data is transmitted at a specific angle over a wide range, resulting in an angle dependent measurement set.

**Figure 8:**
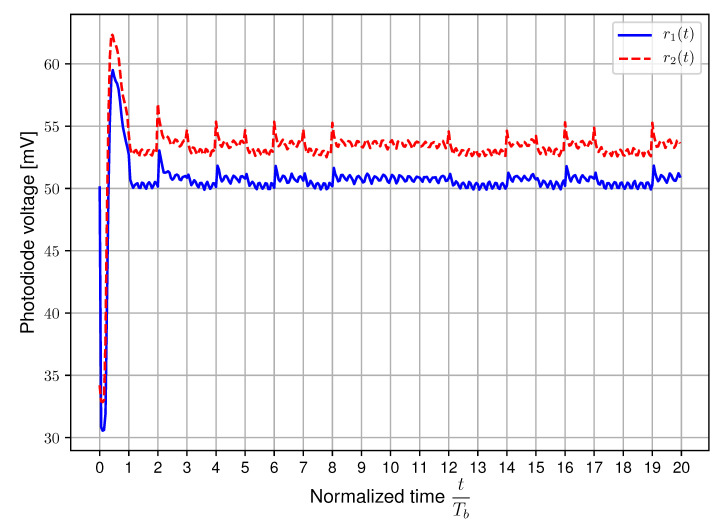
Measured photodiode voltages without receiver polarizers for bit period $T_{b}=250\;\mu$s using setup A [17].

First of all, the light intensity is evaluated without any receiver polarizers equipped, while the LEDs are transmitting data to verify the stability of the DC light level. The results can be seen in Figure 8 for transmitted data with bit period $T_{b}=250\;\mu$s. The remaining noticeable modulated signal is attributable to the deviation of the LED drive currents, but this could be eliminated or reduced by more accurately matching the drive currents.

### 4.1. Setup A

**Figure 9:**
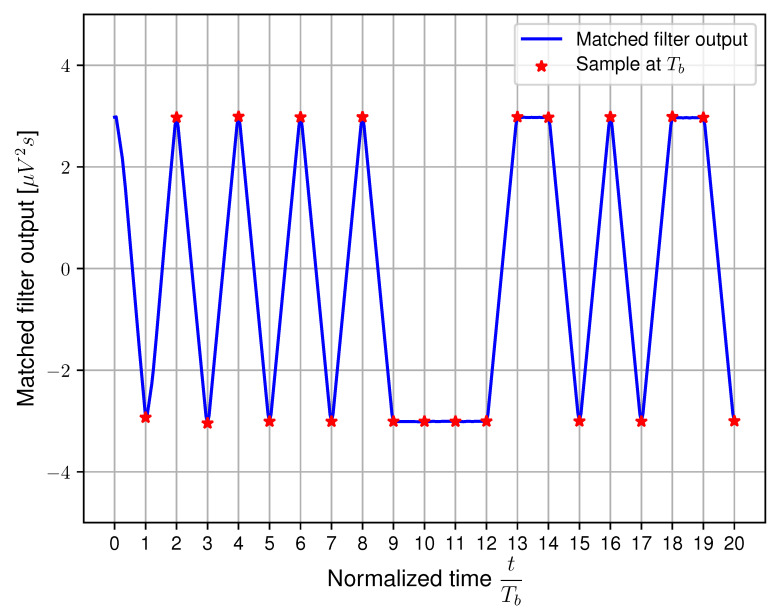
Illustration of the matched filter output for $T_{b}=250\;\mu$s and $\theta =0{\;}^{\circ }$ using setup A [17].

The data transmission and SNR are evaluated with receiver polarizers equipped for setup A by applying matched filtering. The output of the matched filter for $T_{b}=250\;\mu$s and $\theta =0{\;}^{\circ }$ is shown in Figure 9.

**Figure 10:**
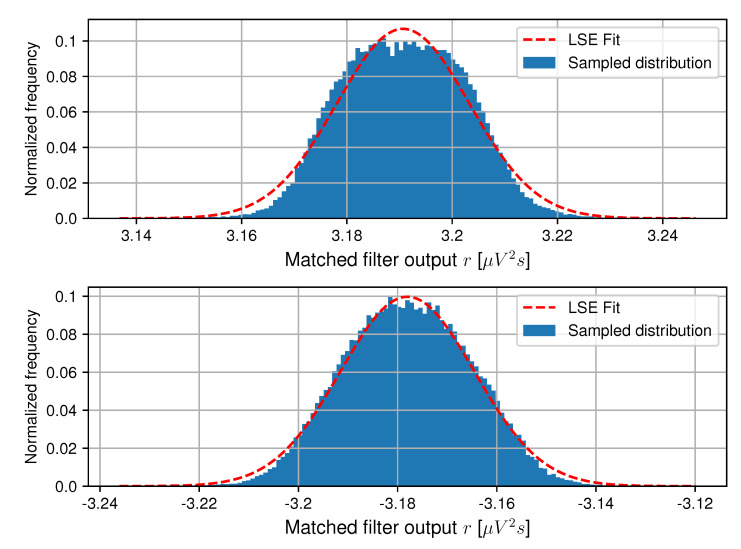
Measured distribution and least square error (LSE) fit of *R* for transmitted ‘1’-bits (top) and ‘0’-bits (bottom) at $\theta =0^{\circ }$ for $T_{b}=250\;\mu$s using setup A [17].

The histogram of the matched filter output distribution *R* is shown in Figure 10 for $\theta =0{\;}^{\circ }$ for both transmitted ‘1’-bits and ‘0’-bits. A Gaussian is fitted to this data using a non-linear least squares error (LSE) fit. This results in $\mu_{R}=3.191\;\mu V^{2}s$ and $\sigma_{R}=0.013\;\mu V^{2}s$ for transmitted ‘1’-bits and $\mu_{R}=-3.178\;\mu V^{2}s$ and $\sigma_{R}=0.013\;\mu V^{2}s$ for transmitted ‘1’-bits. The small deviation between the mean values is most likely attributable to slight variations among the LED drivers.

**Figure 11:**
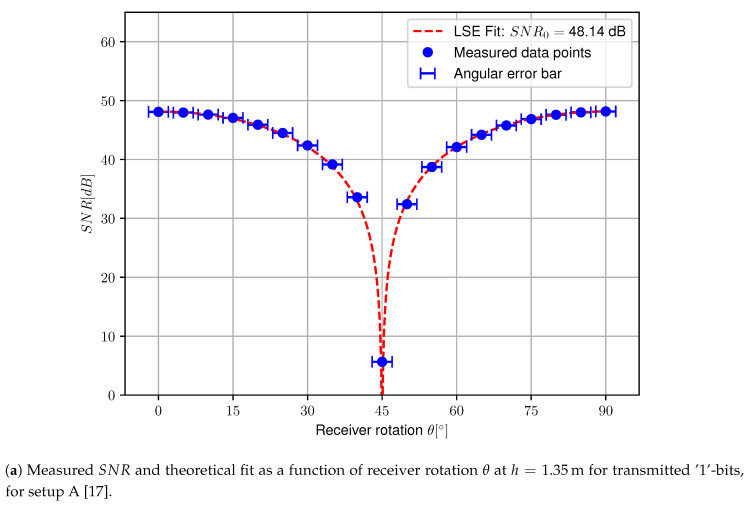

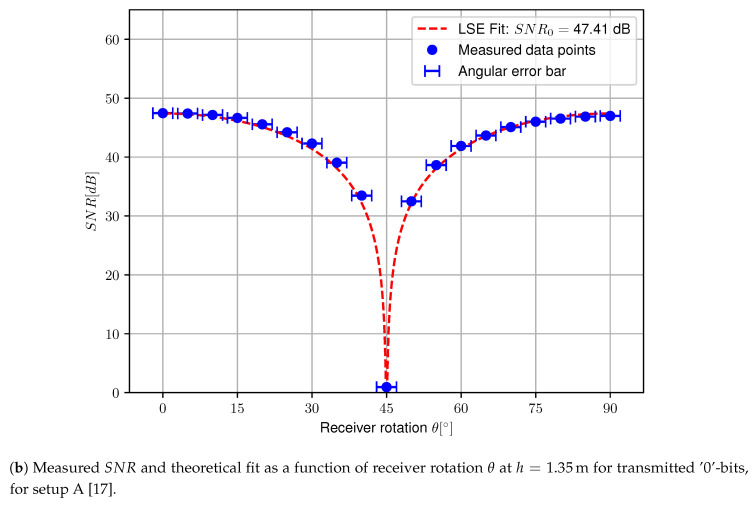
Measured SNR and theoretical fit as a function of receiver rotation θ at h=1.35m for transmitted ‘1’-bits (top) and ‘0’-bits (bottom), for setup A [17].

Finally, the SNR can be calculated according to Equation (10) using the LSE fitted values for $\mu_{R}$ and $\sigma_{R}$. The measured SNR for setup A as a function of the receiver orientation θ is plotted in Figure 11 for transmitted ‘1’- and ‘0’-bits along with $\pm 2^{\circ }$ error bars representing a possible systematic error due to the polarization axis tolerance. The derived model is fitted to this data, resulting in $SNR_{0}=48.14$ dB and $SNR_{0}=47.41$ dB for transmitted ‘1’- and ‘0’-bits respectively. Please note that the logarithmic function plot progresses steeply at $\theta =45{\;}^{\circ }$ as predicted in Section 2.3, hence a minor rotation has a huge impact on the SNR near $\theta =45{\;}^{\circ }$.

Bit-Error Rates can also be predicted based on the measured distributions of the matched filter outputs using Equation (14) [22]. However, as the SNR is significantly high in this setup, the numerical solutions of this equation result in 0, except at $\theta =45{\;}^{\circ }$, where the predicted BER based on the measured distributions of *R* equals $8\times 10^{-2}$. This again corresponds to intuition due to the symmetry at $\theta =45{\;}^{\circ }$, which causes $T_{X1}$ and $T_{X2}$ to be indistinguishable. This predicted value is still considerably better than the theoretical expected BER of 0.5 as a minor misalignment has a significant impact on SNR. Please note that no error correction was applied in the executed measurements. In less favourable conditions, such as lower transmit power or greater link distance, the SNR can drop considerably compared to the presented measurements. In this case, the BER will rise meaning bit errors are much more likely to occur and error correction or detection is essential.

### 4.2. Setup B

**Figure 12:**
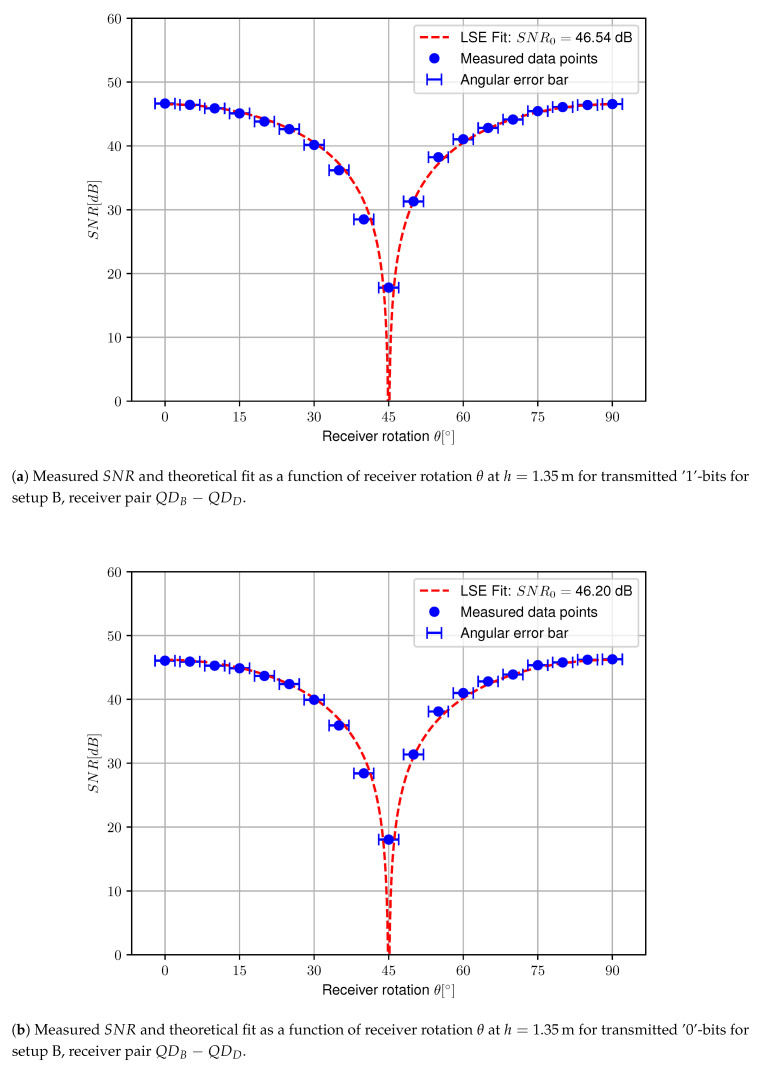

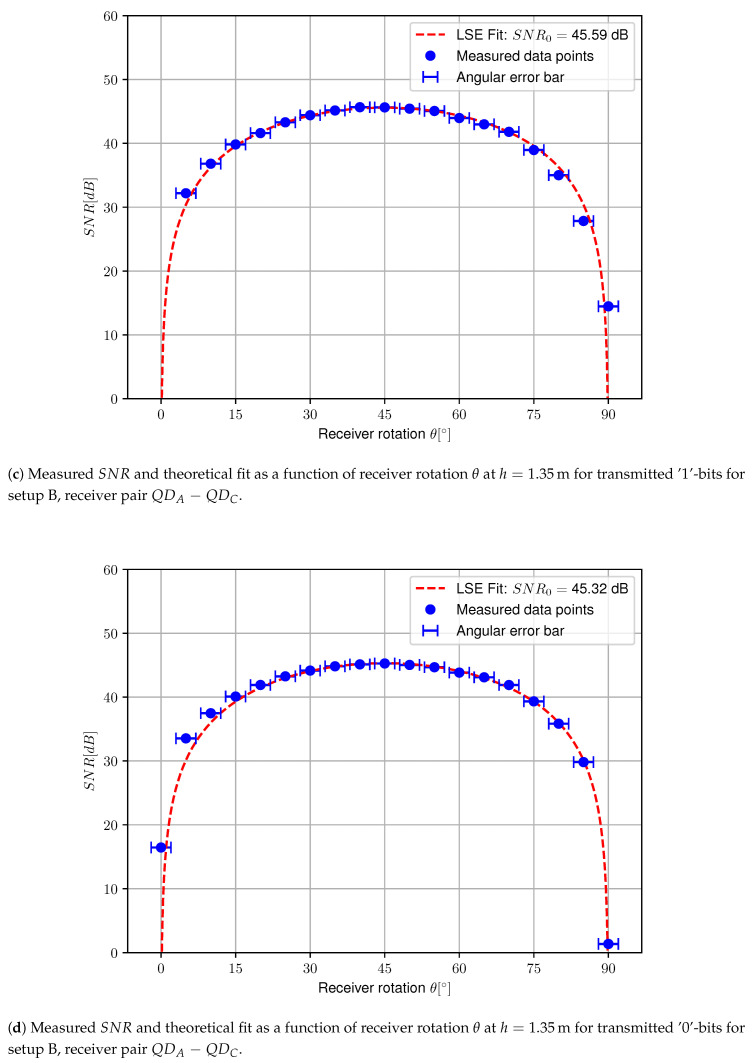
Measured SNR and theoretical fit as a function of receiver rotation θ at h=1.35m for both transmitted ‘1’-bits and ‘0’-bits for both receiver pairs used in setup B.

**Figure 13:**
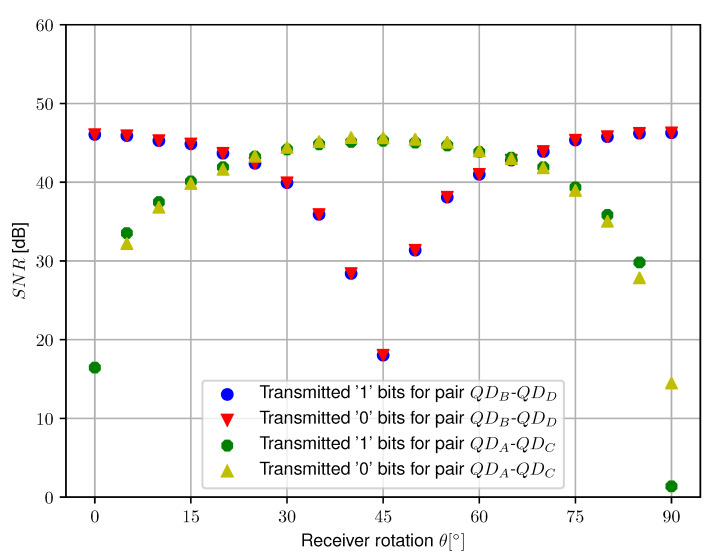
Measured SNR as a function of receiver rotation θ at h=1.35m for setup B.

The same procedure as for setup A is repeated with setup B to assess the SNR for the quadrature receiver. The resulting graphs are plotted in Figure 12, also including an angular error of $\pm 2^{\circ }$ considering the deviation on the polarization axis for the used polarizers. The measured function for the $QD_{B}$−$QD_{D}$ receiver pair progresses similarly to the receiver pair used in setup A. The LSE fit results in $SNR_{0}=46.54$ dB and $SNR_{0}=46.20$ dB for transmitted ‘1’- and ‘0’-bits respectively. At $\theta =45^{\circ }$, the measured SNR severely drops to 17.79 dB and 18.02 dB respectively. In theory, no communication would be possible for this angle as Equation (11) drops to zero, yet a small deviation in angle from the intended $\theta =45^{\circ }$ has an immense impact on the measured SNR. Nevertheless, a severe decrease in SNR occurs for this angle, which can be resolved by switching to the second differential receiver pair $QD_{A}$−$QD_{C}$ for signal demodulation. For this receiver pair, the LSE fit on the measured data points results in $SNR_{0}=45.59$ dB and $SNR_{0}=45.32$ dB for transmitted ‘1’- and ‘0’-bits respectively. Please note that the $SNR_{0}$ derived by LSE fit is about 1 dB lower for this receiver pair, this is most probably caused by a combination of inequalities in transimpedance gain, quadrant responsitivity and small differences in aperture shadowing. Similar to the $QD_{B}$−$QD_{D}$ receiver pair at $\theta =45^{\circ }$, the SNR for receiver pair $QD_{A}$−$QD_{C}$ greatly decreases at $0^{\circ }$ and $90^{\circ }$. This is however not an issue, as receiver pair $QD_{B}$−$QD_{D}$ can be used for demodulation at these angles. Figure 13 illustrates the determined SNR for all data points measured. It is clear that robustness to receiver orientation can be achieved by selecting the pair with the highest SNR for demodulation. The theoretical BER is again determined. For pair $QD_{A}$−$QD_{C}$, this results in a BER of 0.40 and 0.56 at $\theta =0{\;}^{\circ }$ and $\theta =90{\;}^{\circ }$ respectively. For pair $QD_{B}$−$QD_{D}$, this leads to a BER of $2.5\times 10^{-15}$ at $\theta =45{\;}^{\circ }$. Please note that for differential receiver pair $QD_{B}$−$QD_{D}$, the measured BER is still considerably low, again indicating that the angle deviates slightly from the intended $45^{\circ }$.

## 5. Conclusions

In this work, a differential polarization modulation scheme using orthogonal polarizers at both the transmitter and receiver end of the VLC link is demonstrated. A general method to evaluate the SNR as a function of the receiver rotation with regard to the transmitters is determined and experimentally confirmed by measurements. It is shown that differential polarization VLC provides a reliable link for indoor communication due to the high SNR. The SNR is however highly dependent on the receiver orientation. As the relative rotation angle nears $45^{\circ }$, the performance of the communication decreases rapidly. It is demonstrated theoretically and proven experimentally that an additional differential receiver pair rotated over $45^{\circ }$ can mitigate this effect drastically by selecting the receiver pair with the highest SNR for demodulation. The maximum attenuation due to unfavorable receiver rotation is in this case lowered by 3 dB. This demonstrates that the proposed modulation scheme certainly has potential to provide an additional means of transmitting information. In cases where the bounds of the receiver orientation are close to $0^{\circ }$ such as in e.g., Vehicle-to-Vehicle (V2V) communication, a single differential receiver pair can suffice. If this is not the case and any receiver rotation is bound to occur, a second receiver pair can resolve this issue. While the received intensity without receiver polarizers is constant over time, this polarization-based modulation scheme allows for parallel operation alongside conventional intensity-based VLC with no interference. Similarly, the differential operation assures intensity-based VLC does not impede on the polarization-based modulation. As part of future work, there are some interesting points that can be improved upon or further researched. This includes increasing the data rate by modifying the hardware, further automating the data acquisition for different angles using stepper motors, and comparing the proposed modulation technique in terms of performance and robustness to intensity-based modulation in different scenarios. Furthermore, the impact of transmitter induced noise components can be further studied to be included in the model. Additionally, the signal processing in this work was executed in post-processing. The performance can be evaluated in real time using dedicated hardware to execute the signal processing, including some important receiver design improvements such as an AC-filter stage and a differential amplifier stage. Finally, complementary polarization-based variants of other conventional modulation schemes such as Phase Shift Keying and Pulse Position Modulation can be evaluated.

## Figures and Tables

**Table 1:** Comparison between both used setups.

	Setup A	Setup B
**Photodetector**	Thorlabs PDA36A2	Hamamatsu S5981
Active Area	2 × $13\;{\mathrm{mm}}^{2}$	4 × $25\;{\mathrm{mm}}^{2}$
Peak Responsitivity	0.65A/W	0.72A/W
Transimpedance Gain	7.5 kΩ	27 kΩ
DC Bias	0V	2.5V
**DAQ**	National Instruments USB-6215	National Instruments USB-6212
Data rate	4 kbps	4 kbps
Sample rate	80kHz	80kHz
ADC input range	$\left[-0.2\;V,+0.2\;V\right]$	$\left[-10\;V,+10\;V\right]$
ADC resolution	16 bit	16 bit

## Abbreviations

The following abbreviations are used in this manuscript:

VLC	Visible Light Communication
SNR	Signal-to-Noise Ratio
LED	Light-Emitting Diode
LC	Liquid Crystal
PDM	Polarization Division Multiplexing
OFDM	Orthogonal Frequency Division Multiplexing
AoA	Angle-of-Arrival
LP	Linear Polarizer
OOK	On-Off Keying
AWGN	Additive White Gaussian Noise
QD	Quadrant
DAQ	Data Acquisition System
LSE	Least Square Error
BER	Bit-Error Rate
V2V	Vehicle-to-Vehicle
